# *Klebsiella pneumoniae* carbapenemase (KPC)-producing *Klebsiella pneumoniae* ST258 isolated from a Japanese patient without a history of foreign travel - a new public health concern in Japan: a case report

**DOI:** 10.1186/s12879-018-3649-9

**Published:** 2019-01-07

**Authors:** Yusuke Ainoda, Kotaro Aoki, Yoshikazu Ishii, Kentaro Okuda, Hitomi Furukawa, Ryo Manabe, Toshinori Sahara, Fukumi Nakamura-Uchiyama, Hitomi Kurosu, Yukiko Ando, Maki Fujisawa, Hitomi Hoshino, Hideki Arima, Kenji Ohnishi

**Affiliations:** 1 Department of Infectious Diseases, Tokyo Metropolitan Health and Hospitals Corporation Ebara Hospital, 4-5-10 Higashi-Yukigaya Ota-ku, Tokyo, 145-0065 Japan; 2 Department of Microbiology and Infectious Diseases, Toho University School of Medicine, 5-21-16 Omori-Nishi Ota-ku, Tokyo, 143-8540 Japan; 3 Department of Pulmonary Medicine, Tokyo Metropolitan Health and Hospitals Corporation Ebara Hospital, 4-5-10 Higashi-Yukigaya Ota-ku, Tokyo, 145-0065 Japan; 4 Department of Laboratory, Tokyo Metropolitan Health and Hospitals Corporation Ebara Hospital, 4-5-10 Higashi-Yukigaya Ota-ku, Tokyo, 145-0065 Japan

**Keywords:** *Klebsiella pneumoniae* carbapenemase (KPC)-producing *Klebsiella pneumoniae*, *K. pneumoniae* ST258, Japanese

## Abstract

**Background:**

Thus far, studies on *Klebsiella pneumoniae* carbapenemase (KPC)-producing organisms have only been reported in those with a history of foreign travel, and a specific Japanese KPC-producing isolate has not yet been reported.

**Case presentation:**

We describe a Japanese patient, with no history of travel to foreign countries, admitted due to aspiration pneumonia, and a KPC-producing isolate detected in his sputum. Fortunately, his pneumonia resolved. His close contacts did not have a history of foreign travel, and the isolate was not detected in other patients.

**Conclusions:**

The potential for KPC-producing organisms to become endemic in Japan is currently of great concern.

## Background

*Klebsiella pneumoniae* carbapenemase (KPC)-producing *Enterobacteriaceae* have been reported in many countries, and outbreaks due to KPC-producing organisms have been reported worldwide [1–3]. KPC is an enzyme that degrades penicillin, cephalosporin, and broad-spectrum β-lactams such as the carbapenems. This type of β-lactamase was discovered relatively recently, first detected in a strain isolated in the United States in 1996 [2]. The strain was considered a novel β-lactamase able to degrade carbapenem antibiotics; it was classified as a Bush group 2f, class A, serine-β-lactamase and was named KPC [4]. Because the mortality from infection by KPC-producing organisms is reportedly higher than that of non-KPC-producers, these isolates have received a great deal of attention worldwide [5]. The most common carbapenemase gene to be reported in Japan is the IMP-type metallo-β-lactamase encoding gene [6] and the most common types are *bla*_IMP-1_ and *bla*_IMP-6_. However, in Japan, KPC-producing organisms have so far only been reported as infections in patients who had visited foreign countries wherein KPC-producing organisms have been reported [7]. We report a Japanese patient without a history of travel to foreign countries whose sputum samples tested positive for a KPC producing isolate. The current report highlights a new concern regarding KPC-producing organisms that may already be present in Japan.

## Case presentation

A man in his early 90s was undergoing a follow-up for mild idiopathic interstitial pneumonia. He required assistance to perform activities of daily living, spent most of the day at home, and received periodic home visits for medical care. His last hospitalization was in February 2016 for approximately 1 month due to aspiration pneumonia. Only oral commensal bacteria were cultured from his sputum during his last hospitalization. In July 2016, he was hospitalized again for aspiration pneumonia. The sputum smears obtained on the first day of admission showed the presence of polymicrobial, normal oral bacteria and polymorphonuclear leukocytes. Subsequent cultures from this sputum showed normal oral bacteria as well as a few *K. pneumoniae,* with high levels of resistance to all antimicrobial agents except for minocycline. Results of examination of blood culture obtained on admission were negative. Other cultures were not examined. The patient had no history of travel to other countries and had never left Japan. Ampicillin/sulbactam was started at the time of hospitalization. On the 4th day of hospitalization, the antimicrobial agent was changed to cefepime because the clinical course was exacerbated. After the 5th day, the patient’s clinical course improved, and this treatment was continued until the 12th day. The antimicrobial was not changed when *K. pneumoniae* was observed on a sputum culture collected on admission. The patient was discharged after his aspiration pneumonia had been successfully treated. Despite administering antibiotics that are generally not effective against *K. pneumoniae*, *K. pneumoniae* was not detected from his sputum after treatment.

### Laboratory investigation

**Fig. 1:**
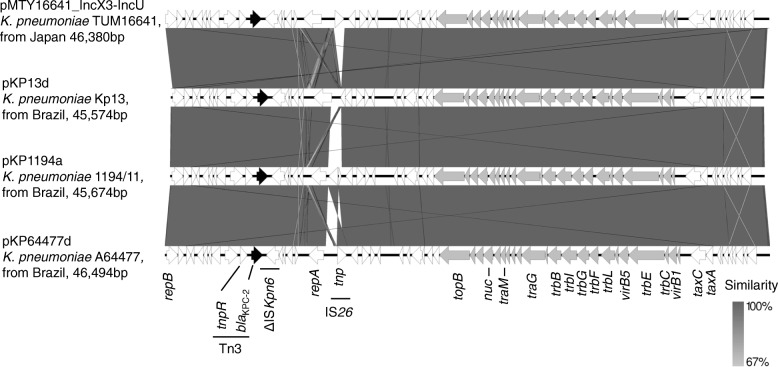
Comparison of pMTY16641_IncX3_IncU (BFCA01000004) carrying *bla*_KPC-2_ with pKP13d (CP003997), pKP1194a (KX756453), and pKP64477d (MF150120) drawn with EasyFig 2.1. All four plasmids were hybrid replicons of IncX3 and IncU. Arrow size is proportional to the predicted ORF length. The color code is as follows: The antibiotic resistance genes are in black and conjugal transfer genes are in gray. Others, putative, hypothetical, and unknown genes are represented by white arrows. Abbreviations: ORF: Open Reading Frame

During laboratory investigation, we found that gram-negative bacillus grew on 5% sheep blood agar. Carbapenem-resistant *K. pneumoniae* was identified by Phoenix100 and NMIC/ID-208 panel (Becton, Dickinson and Company). Minimum inhibitory concentration of both meropenem and imipenem was > 8 μg/ml, and the sodium mercaptoacetate disk test result was negative. The modified Hodge test (using ertapenem disk) result was positive for *K. pneumoniae* TUM16641. The DNA of *K. pneumoniae* TUM16641 was sequenced using MiSeq (Illumina, Inc., CA, USA), and the DNA library for Illumia MiSeq sequencing was prepared using the Nextera XT Library Prep Kit (Illumina). The Nextera XT DNA library was sequenced in a paired-end 300 cycles mode on MiSeq using 600 cycles Reagent Kit v3 (Illumina). Draft genomes (contigs) were obtained using CLC Genomics Workbench (Qiagen). TUM16641 belonged to sequence type (ST) 258 analyzed by multilocus sequence typing. A carbapenemase gene, *bla*_KPC-2_, was detected in the contigs. To characterize a *bla*_KPC-2_ carrying plasmid, we used a long reads sequencing platform, MinION (Oxford Nanopore Technologies [ONT], Oxford Science Park, UK). A MinION library was prepared from *K. pneumoniae* TUM16641 genomic DNA using Ligation Sequencing Kit 1D (SQK-LSK108) and Native Barcoding Kit (EXP-NBD103) (ONT). The MinION DNA library was sequenced using Flow Cell R9.4 (FLOW-MIN106) (ONT). The complete plasmid sequence was obtained using SPAdes assemblers in combination with MiSeq and MinION data [8]. The sequencing data showed that the *K. pneumoniae* TUM16641 harbored a hybrid replicon of the IncX3 and IncU plasmid (pMTY16641_IncX3-IncU) carrying *bla*_KPC-2_ (Fig. 1). The nucleotide sequence of pMTY16641_IncX3-IncU plasmid (GenBank accession number BFCA01000004) highly resembled that of pKP13d, pKP1194a, and pKP64477d of *K. pneumoniae* obtained from different reports in Brazil (Fig. 1). *K. pneumoniae* TUM16641 also harbored two antibiotic resistance gene carrying plasmids, a hybrid replicon of IncFIB and IncFII plasmid (pMTY16641_IncFIB-IncFII) carrying *aadA2*, *aph(3′)-Ia*, *mph(A)*, *catA*, *sul1*, and *dfrA12* and a IncA/C2 plasmid (pMTY16641_IncA/C2) carrying *aac(3′)-IId*, *rmtB*, *strA*, *strB*, *bla*_TEM-1B_, *bla*_CTX-M-14_, *sul2*, *tet(G)* (Table 1).

**Table 1:** *K. pneumoniae* harboring replicons (chromosome and plasmid) and antibiotic resistance genes

Replicon and plasmid Inc/Rep type	Antibiotic resistance genes
Chromosome	*bla*_SHV-11_, *oqxA, oqxB, fosA,*
IncFIB and IncFII plasmid	*aadA2, aph(3′)-Ia, mph(A), catA, sul1, dfrA12*
IncA/C2 plasmid	*aac(3′)-IId, rmtB, strA, strB*, *bla*_TEM-1B_^a^, *bla*_CTX-M-14_*, sul2, tet(G)*
IncX3 and IncU plasmid	*bla*_KPC-2_
ColRNAI plasmid	ND

^a^Two *bla*_TEM-1B_ were located on IncA/C2 plasmid. ND: not detected

The GenBank accession number for the draft whole-genome sequence data of the *K. pneumoniae* TUM16641 is DRR076334.

## Discussions and conclusions

To our knowledge, this case showed the first possibility of a new threat in Japan regarding a KPC-producing isolate not linked with a history of travelling to other countries. The KPC-producing isolate was not detected in any other patients in the hospital where this patient was hospitalized previously; thus, the transmission route of this strain remains unclear. However, the present case suggests that KPC-producing organisms may be prevalent in Japan. Although no environmental culture was performed, the same bacteria were not observed in general culture tests of other patients who were hospitalized in the same medical institution. The origin of this isolate was unclear. This isolate was similar to a type reported in Brazil (assigned to the GenBank nucleotide sequence database under the accession number KU963389.1) and the USA [9]. The reported risks for infection by KPC-producing bacteria include patient immunodeficiency, use of intravascular devices, and use of various antimicrobial agents [10, 11]. No intravascular devices and no agents that could lead to immunodeficiency were used in this patient. However, considering that he was elderly, had experienced aspiration pneumonia, and had undergone 10 days of antibiotic treatment 5 months previously, the KPC-producing organism identified in his sputum may have been selected by exposure to ampicillin/sulbactam and meropenem. This patient’s symptoms were relieved by treatment of his bacterial pneumonia. The patient was likely a carrier of a KPC-producing isolate, as he did not develop a *K. pneumoniae* infection*.* Clinical improvement without susceptible antimicrobial agents suggests the possibility that *K. pneumoniae* was not the causative organism in the present case.

We report a new public health concern in Japan regarding a KPC-producing isolate detected in a Japanese patient with no history of foreign travel. These findings highlight a growing concern regarding the potential spread of KPC-producing organisms, and that the possibility of detecting such bacteria should be considered even in Japan.

## Abbreviation

KPC: *Klebsiella pneumoniae* carbapenemase

## References

[1] Naas T, Nordmann P, Vedel G, Poyart C (2005). Plasmid-mediated carbapenem-hydrolyzing β-lactamase KPC in a *Klebsiella pneumoniae* isolate from France. Antimicrob Agents Chemother.

[2] Woodford N, Tierno PM, Young K, Tysall L, Palepou MF, Ward E (2004). Outbreak of *Klebsiella pneumoniae* producing a new carbapenem-hydrolyzing class a beta-lactamase, KPC-3, in a New York medical center. Antimicrob Agents Chemother.

[3] Wei ZQ, Du XX, Yu YS, Shen P, Chen YG, Li LJ (2007). Plasmid-mediated KPC-2 in a *Klebsiella pneumoniae* isolate from China. Antimicrob Agents Chemother.

[4] Yigit H, Queenan AM, Anderson GJ, Domenech-Sanchez A, Biddle JW, Steward CD (2001). Novel carbapenem-hydrolyzing β-lactamase, KPC-1, from a carbapenem-resistant strain of *Klebsiella pneumoniae*. Antimicrob Agents Chemother.

[5] Marchaim D, Navon-Venezia S, Schwaber MJ, Carmeli Y (2008). Isolation of imipenem-resistant *Enterobacter* species: emergence of KPC-2 carbapenemase, molecular characterization, epidemiology, and outcomes. Antimicrob Agents Chemother.

[6] Yano H, Ogawa M, Endo S, Kakuta R, Kanamori H, Inomata S (2012). High frequency of IMP-6 among clinical isolates of metallo-β-lactamase-producing *Escherichia coli* in Japan. Antimicrob Agents Chemother.

[7] Saito R, Takahashi R, Sawabe E, Koyano S, Takahashi Y, Shima M (2014). First report of KPC-2 carbapenemase-producing *Klebsiella pneumoniae* in Japan. Antimicrob Agents Chemother.

[8] Bankevich A, Nurk S, Antipov D, Gurevich AA, Dvorkin M, Kulikov AS (2012). J Comput Biol.

[9] Ho PL, Cheung YY, Lo WU, Li Z, Chow KH, Lin CH (2013). Molecular characterization of an atypical IncX3 plasmid pKPC-NY79 carrying *bla*_KPC-2_ in a *Klebsiella pneumoniae*. Curr Microbiol.

[10] Nordmann P, Cuzon G, Naas T (2009). The real threat of *Klebsiella pneumoniae* carbapenemase-producing bacteria. Lancet Infect Dis.

[11] Woodford N, Tierno PM, Young K, Tysall L, Palepou MF, Ward E (2004). Outbreak of *Klebsiella pneumoniae* producing a new carbapenem-hydrolyzing class a β-lactamase, KPC-3, in a New York medical center. Antimicrob Agents Chemother.

